# Carbon Nanodots as Peroxidase Nanozymes for Biosensing

**DOI:** 10.3390/molecules21121653

**Published:** 2016-12-02

**Authors:** Bhaskar Garg, Tanuja Bisht

**Affiliations:** 1 Department of Chemistry, Indian Institute of Technology Roorkee, Roorkee, Uttarakhand 247667, India; 2 Department of Chemistry, Government Post Graduate College, Champawat, Uttarakhand 262523, India; tanuja.bisht16@gmail.com

**Keywords:** carbon nanodots, carbon quantum dots, graphene quantum dots, peroxidases, enzyme mimetics, photoluminescence, biosensors, tetramethylbenzidine, carbon nitride dots

## Abstract

‘Nanozymes’, a term coined by Scrimin, Pasquato, and co-workers to describe nanomaterials with enzyme-like characteristics, represent an exciting and emerging research area in the field of artificial enzymes. Indubitably, the last decade has witnessed substantial advancements in the design of a variety of functional nanoscale materials, including metal oxides and carbon-based nanomaterials, which mimic the structures and functions of naturally occurring enzymes. Among these, carbon nanodots (C-dots) or carbon quantum dots (CQDs) offer huge potential due to their unique properties as compared to natural enzymes and/or classical artificial enzymes. In this mini review, we discuss the peroxidase-like catalytic activities of C-dots and their applications in biosensing. The scope intends to cover not only the C-dots but also graphene quantum dots (GQDs), doped C-dots/GQDs, carbon nitride dots, and C-dots/GQDs nanocomposites. Nevertheless, this mini review is designed to be illustrative, not comprehensive.

## 1. Introduction

‘Artificial enzymes’, a term invented by Ronald Breslow [1], is an stimulating branch of biomimetics, which relies on the use of alternative molecules or materials to either reproduce the elusive structure of enzyme active sites or mimic the functions of the natural enzymes [2,3]. ‘Nanozymes’—nanomaterials with enzyme-like characteristics—represent an emerging research area in the field of artificial enzymes and have generated enormous scientific excitement because of their superiority in terms of elegant response to external stimuli, self-assembly capability, large surface area, ease of desirable yet diversified structural modulations or bioconjugation, and most importantly, size-dependent catalytic activities [4,5]. Not surprisingly, therefore, the past few years have witnessed the exploration of a variety of nanomaterials to mimic the structures and functions of natural enzymes, including, but not limited to, peroxidase (POD), catalase, oxidase, superoxide dismutase, phosphatase, and nuclease for diverse applications [5,6,7,8,9].

Carbon, is the 15th most abundant natural element found in the Earth’s crust. Carbon is abundant in the Sun, stars, comets, and in the atmosphere of most planets. This abundance, combined with the exceptional but unfamiliar polymer-forming ability of organic compounds at Earth’s temperatures makes carbon the chemical basis of all known life. The carbon allotropes such as amorphous carbon or coal, graphite and diamond have found many usages over the centuries. From providing energy, to being the most expensive crystal (a sensation among female fans), to support a central need in education, giving us pencils, a tool for many; carbon has done it all. Recently, with the rapid rise of classical nanofabrication techniques, the carbonaceous nanomaterials such as carbon nanotubes (CNTs) and graphene are (and will continue to be) increasingly used in the imminent carbon age for a diverse range of catalytic applications, including enzyme mimics [10,11,12,13,14,15,16,17].

**Figure 1:**
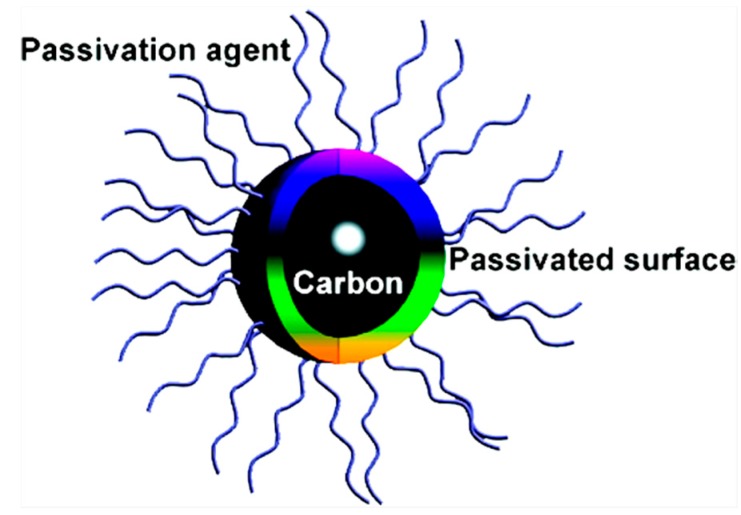
Schematic illustration of the structure of C-dots. Reproduced with permission from [19]. Copyright (2010) American Chemical Society.

Since their initial discovery in 2004 [18], zero-dimensional carbon nanodots (C-dots) or carbon quantum dots (CQDs) have gradually become a rising star as a new member in the family of carbonaceous nanomaterials and the scope of their utility has increasingly matured in the last few years for variety of applications such as biosensing, bioimaging, and drug delivery and therapy [19,20,21,22,23]. Owing to their strong luminescence, in contrast to macroscopic carbon, C-dots are also referred to as nanolights or fluorescent carbon. In general, C-dots are quasi-spherical carbon nanoparticles (C-NPs) that comprise amorphous to nanocrystalline cores with a diameter of less than 10 nm. A representative structure of C-dots is depicted in Figure 1.

It has been demonstrated that the inner part of C-dots is primarily composed of sp^2^-hybridized carbon atoms while sp^3^-hybridized carbon atoms are present in the outer part, as inferred from nuclear magnetic resonance (NMR) measurements [24]. Similar to its predecessors, the semiconductor quantum dots (QDs), C-dots also exhibit an excellent electrochemiluminescence emission property. Nevertheless, the exceptional physicochemical properties such as an excellent water dispersibility (thanks to the abundant hydrophilic groups on their surface), robust chemical inertness, low toxicity, good biocompatibility, high resistance to photobleaching, ease of synthesis, excitation-dependent multicolor emission, and desirable surface engineering with all facets of C-dots make their profile a collection of very desirable and remarkable traits, which enable them as the nanomaterials-of-choice for enzymatic applications, in particular, a POD mimics.

**Scheme 1:**
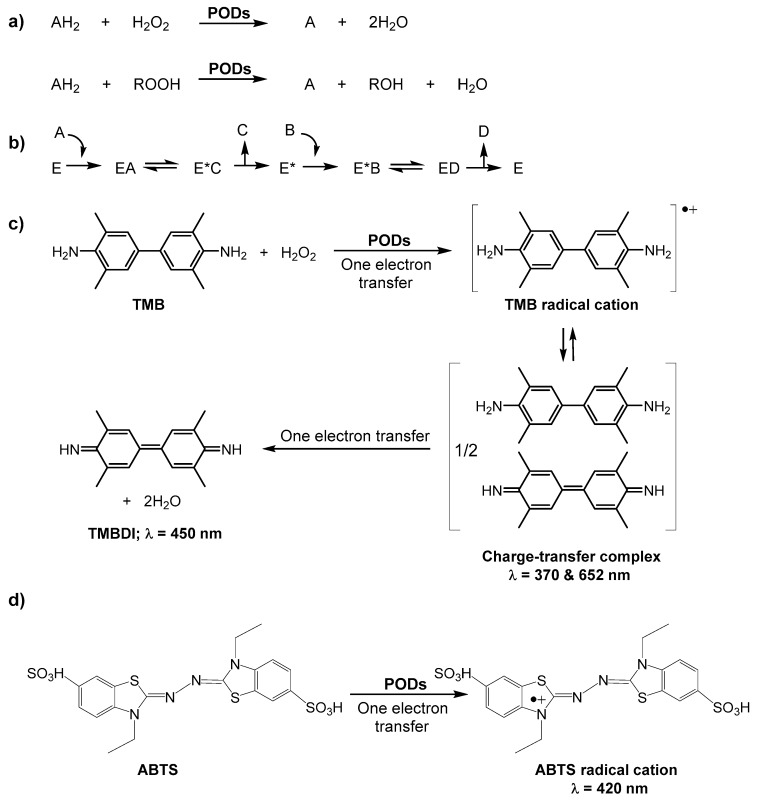
(**a**) Representative reactions catalyzed by PODs; (**b**) a schematic for ping-pong mechanism; (**c**) schematic for POD-catalyzed two electron oxidation of TMB into TMBDI and (**d**) schematic for POD-catalyzed one electron oxidation of ABTS.

PODs (EC 1.11.1.7) or a group of oxidoreductases are a family of isoenzymes that play a critical role in different metabolic activities in living organisms. Typically, the catalytic reactions of PODs involve the oxidation of an electron-donor substrate with the simultaneous reduction of H_2_O_2_ or occasionally alkyl hydroperoxide (Scheme 1a). Horseradish peroxidase (HRP) containing the heme-iron cofactor is the most abundant POD isoenzyme, which functions like a ping-pong ball, bouncing back and forth from an intermediate state (E*) to its standard state (E). Consequently, one substrate (A) converts to the product (C) and releases before the second substrate (B) binds to the enzyme. The overall process of this double displacement reaction is highlighted in Scheme 1b. The POD-like activity of a desired material, for example, C-dots, can be evaluated by measuring the concentration of H_2_O_2_ using a variety of chromogenic substrates such as hydroquinone, 1,2,3-trihydroxybenzene (THB) or pyrrogallol, *o*-phenylenediamine (OPD), 2,2’-azino-bis-(3-ethylbenzothiozoline-6-sulfonic acid (ABTS), and 3,3’,5,5’-tetramethylbenzidine (TMB). However, owing to the low carcinogenicity and high absorption coefficient of its reaction products, TMB serves as the most-studied chromogen for HRP mimics, in acidic conditions. In general, the POD-catalyzed oxidation of TMB + H_2_O_2_ system involves two electron transfer steps. In the first step, a one electron oxidation generates a TMB radical cation. Two of these intermediate radical cations then form a blue-colored charge-transfer complex (λ = 370 & 652 nm), a diagnostic for HRP mimic, as shown in Scheme 1c. The cation radical is further oxidized in the second electron transfer step, producing diimine derivative of TMB (TMBDI; λ = 450 nm). That is why; the absorption spectrum of the oxidation product of TMB usually exhibits three absorption bands. ABTS is yet another important chromogen in POD reactions, which typically produces green color due to the formation of ABTS radical cation in the presence of H_2_O_2_. The one electron transfer step in the oxidation of ABTS is shown in Scheme 1d.

Very recently we have provided an authoritative detail and comprehensive account on the applications of graphene-based nanomaterials as versatile catalysts in POD-mimicking reactions [16]. In this mini review, we will provide a systematic and organized survey of C-dots as nanozymes with POD-like activities as well as their applications in biosensing. We anticipate that the years’ efforts in developing C-dots as potential POD-mimic have not been covered by any research group in a review format and yet to be published. The scope intends to cover not only the C-dots but also graphene quantum dots (GQDs), doped C-dots/GQDs, C-dots/GQDs nanocomposites, and carbon nitride dots (CN-dots). However, as the synthesis and properties of C-dots have been comprehensively reported elsewhere [20,21,22,23,25], we will just briefly discuss the fundamentals of the same. Furthermore, readers are recommended to consult other potential review articles for the corresponding unique and productive details on POD enzymes as well as nanozymes [5,8,16,26,27,28]. It is our hope that this mini review will provide a valuable insight and stimulate researchers for the development of better performing C-dots (in terms of good water dispersibility, (photo)stability and cell permeability) as nanozymes.

## 2. Fundamentals of C-Dots: An Overview

**Figure 2:**
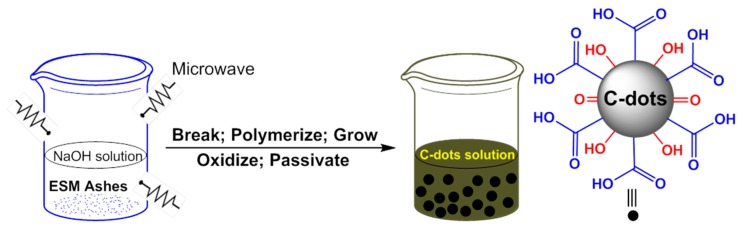
Schematic illustration of a MW-assisted synthesis of C-dots. Adapted with permission from [30]. Copyright (2012) Royal Society of Chemistry.

Given the size-controllable, handy, cost-effective, eco-friendly, and large scale synthesis of C-dots, a variety of synthetic routes have been developed during the last decade. Most of these routes can be broadly categorized into ‘Top-down’ and ‘Bottom-up’ methods. Typically, in the ‘Top-down’ approaches, C-dots are prepared by means of breaking down bulk carbon materials such as CNTs, graphene and suspended C-powders [29], to highlight a few. Laser irradiation, electrochemical carbonization, plasma treatment and arc discharge are the routinely employed ‘Top-down’ approaches. On another front, in the ‘Bottom-up’ approaches, an organic precursor or molecular carbon sources such as sucrose, citric acid and amino acids are used as seeds to generate C-dots under certain experimental conditions. Hydrothermal or solvothermal carbonization, ultrasonic synthesis and microwave (MW)-assisted synthesis of C-dots can be recapitulated under ‘Bottom-up’ approaches. Among these, MW-assisted synthesis of C-dots is considered as an energy efficient and eco-friendly process which can shorten the reaction time significantly, a factor than can dominate over ultrasonic synthesis. In their pioneering work, Lv and co-workers [30] first reported the use of MW technology for the synthesis of C-dots from the ashes of egg-shell membrane (ESM), a protein-rich waste. The typical schematic illustration for the MW-assisted formation of C-dots is highlighted in Figure 2.

C-dots are chiefly composed of carbon, oxygen, hydrogen and nitrogen atoms, and the ratio of these elements can vary significantly in raw and purified C-dots. For instance, the chemical composition of the raw candle soot (91.7% C, 1.8% H, 1.8% N, 4.4% O) has been found hugely different from that of purified C-dots (36.8% C, 5.9% H, 9.6% N, 44.7% O) [24]. Nevertheless, most of the purified C-dots contain oxygen as the abundant element due to the presence of oxygen-containing functional groups, rendering them hydrophilic in nature. Aside from that of elemental analysis, C-dots can be conveniently characterized by high resolution transmission electron microscopy (HRTEM), atomic force microscopy (AFM), X-ray diffraction (XRD), Fourier transform infrared spectroscopy (FTIR), X-ray photoelectron spectroscopy (XPS), UV-visible and fluorescence spectroscopy, thermogravimetric analysis (TGA), and ^13^C solid state NMR measurements [31].

Most C-dots usually exhibit a typical absorption band around 260–320 nm, which may shift to longer wavelengths after surface passivation with amino or silane compounds. It is worthwhile to mention here that despite the elusiveness of its mechanism, surface passivation significantly affects the photoluminescence (PL) of C-dots and appears to be associated with the synthetic method used. The PL emission of C-dots, including both the excitation wavelength-dependent and independent, is one of the most appealing features and can be used to manipulate the photoinduced electron transfer [32] and thus may pave the way for novel applications in energy conversion. Although the exact mechanism(s) of the PL of C-dots are still a matter of debate, some aspects are well recognized. It is speculated that recombination of excitons, quantum effect (due to the different-sized NPs, and/or the different emissive traps on the surface of C-dots significantly contribute to the phenomenon [20]. A closely related issue, which has drawn researchers’ attention, is the quantum yield (QY), the classical signature of the PL properties of C-dots. To increase the QY, several approaches such as surface passivation, inorganic salt or element doping, and purification procedures have been surfaced from time to time and are significantly influencing the current trends in the biological applications of C-dots. For instance, P-doped C-dots are found to exhibit green fluorescence and could be used as imaging signal with low background [33]. At this juncture, it is critical to point out that nitrogenous reagents can serve as both N-source and surface passivator and, thus create complexity in differentiation between N-doping and passivation [34]. 

The low toxicity and biocompatibility of C-dots both in vitro and in vivo are other intriguing properties, making them suitable for biological applications. It has been found that there is direct correlation between the surface passivation agents and toxicity of C-dots. Specifically, C-dots functionalized with less cytotoxic passivating agents such as polyethylene glycol (PEG) and poly-(propionylethylenimine-co-ethylenimine) (PPEI-EI) usually generate C-dots with low cytotoxicity [35].

## 3. Carbon Nanodots (C-Dots) as POD Nanozymes

**Figure 3:**
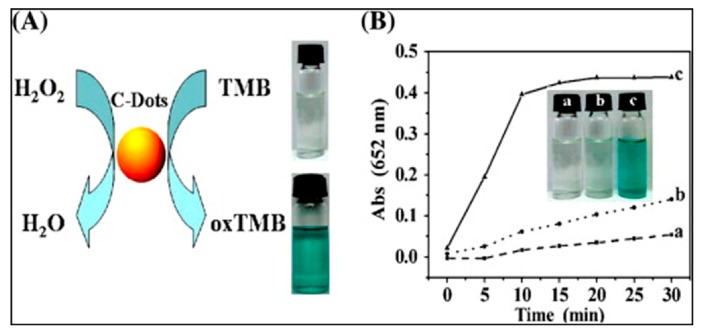
(**A**) Schematic illustration of oxidation color reaction of TMB by H_2_O_2_ catalyzed by C-dots; (**B**) Time-dependent absorbance changes at 652 nm of TMB in different reaction systems: (a) C-dots + TMB, (b) TMB + H_2_O_2_ and (c) TMB + C-dots + H_2_O_2_ in sodium acetate buffer (0.2 M) at pH 3.5 and a temperature of 35 °C. Reproduced with permission from [36]. Copyright (2011) Royal Society of Chemistry.

In pioneering work, Zheng, Huang and co-workers [36] discovered that C-dots from candle soot can exhibit POD-like catalytic activity. In particular, in the presence of H_2_O_2_ in sodium acetate buffer, C-dots could catalyze the three POD substrates, namely, TMB, OPD and THB, producing typical color reactions (Figure 3). It was concluded that the catalytic mechanism is probably due to an increase in the electron density and mobility in the C-dots due to the electron transfer from TMB to C-dots. This results in an acceleration of electron transfer from C-dots to H_2_O_2_ and thus increase the rate of TMB oxidation. Under the optimized conditions (pH 3.5, 35 °C, and 300 mM H_2_O_2_), C-dots exhibited maximum catalytic activity, which was strongly dependent on different conditions such as pH, temperature, and H_2_O_2_/C-dots concentration, similar to that of HRP. Nevertheless, in contrast to HRP, the catalytic activity of C-dots remained stable over a wide pH range (2–12) as well as temperature (0–90 °C). The kinetic data revealed that C-dots has higher affinities to H_2_O_2_ and POD substrates and display a ping-pong mechanism, similar to HRP.

Glucose oxidase (GOx) also known as notatin (EC 1.1.3.4) is a dimeric oxido-reductase protein, widely used in POD reactions in the determination of free glucose in blood plasma for diagnostics. By combining the POD-like activity of C-dots with GOx, a simple, cheap, selective, and sensitive colorimetric assay was developed to detect glucose in serum samples [36].

In parallel with this work, Qu and co-workers [37] also prepared C-dots from candle soot and examined their POD-like activity. In contrast to HRP, CNTs, graphene oxide (GO) and Fe_3_O_4_ NPs, the catalytic activity of C-dots was found to increase with increasing temperature with activation energy of ca. 59.3 kJ/mol. Based on the intrinsic enzyme activity of C-dots, a quantitative assay was developed for colorimetric detection of glucose in real samples, such as diluted blood and fruit juice. Aside from their intrinsic POD-like activity, C-dots can act as electron donors and transporters, and were successfully used to form C-dots-porphyrin supramolecular composites by making the use of electrostatic and π-stacking interactions.

**Figure 4:**
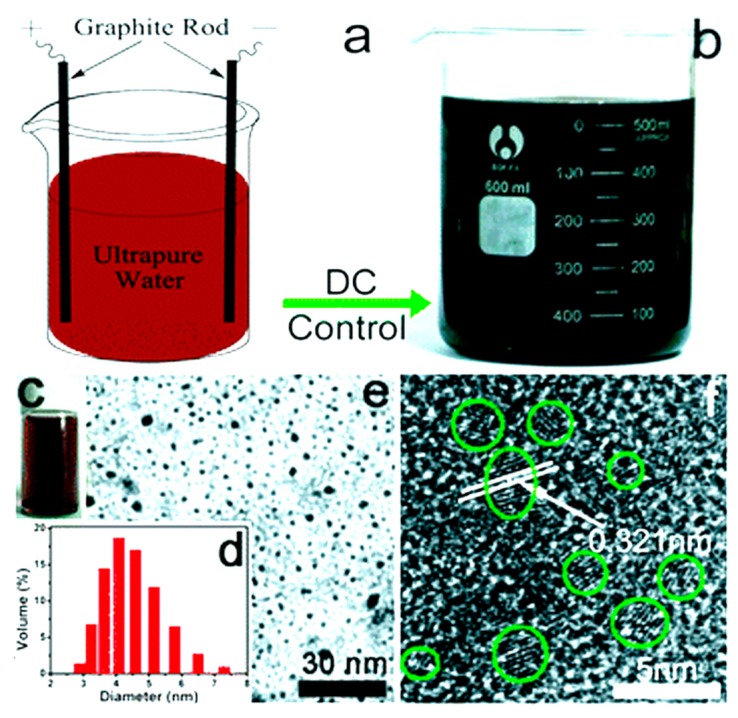
(**a**) Reaction equipment for the preparation of C-dots; digital image of C-dots solution (**b**) before treatment; (**c**) after treatment; (**d**) DLS histogram of C-dots; (**e**) TEM; and (**f**) HRTEM image of C-dots. Reproduced with permission from [38]. Copyright (2012) Royal Society of Chemistry.

These two initial studies set the new directions and opened the door for the development of a variety of C-dots as well as their composites as POD nanozymes. Liu, Kang and co-workers [38] relied on the use of an eco-friendly, cost-effective, and additive-free one step electrochemical approach for the preparation of C-dots (3–6 nm), without the assistance of any chemicals but only ultra-pure water in the reaction system (Figure 4). The as-prepared C-dots exhibited remarkable PL properties and mimicked POD functions in the degradation of methyl orange (MO). Upon mixing H_2_O_2_ and MO (pH 7.0) with C-dots, only a moderate degradation of MO (23.6%, 160 min) was recognized. However, a better catalytic activity was achieved in the presence of visible light and MO could be photo-decomposed completely within 80 min. Following a hydrothermal route, the protocol was further extended to fabricate a hybrid photocatalyst (TiO_2_/C-dots) with an excellent visible-light-driven photocatalytic activity.

Safavi and co-workers [39] introduced a MW-assisted ionic liquid (MW-IL) method to generate C-dots with narrow size distribution. The POD-like activity of as-prepared C-dots was examined in the degradation of methyl red (MR) and MO as model azo dyes. The respective value of the rate constant and degradation efficiency of the dye were estimated to be 9.5 × 10^−3^ min^−1^ and 83% after 200 min, which suggest a good catalytic activity of C-dots. These studies [38,39] may pave the way for C-dots applications in the versatile areas of immunohistochemistry, photocatalysis, and environmental science.

In subsequent work(s), the group further utilized the intrinsic POD activity of C-dots in the indirect and direct colorimetric detection of glutathione (GSH) and mercury ion [40,41], respectively. In contrast to GSH (a detection limit of 0.3 μM), a limit of detection for mercury ion was estimated to be 23 nM, with tunable dynamic range.

A facile and cost-effective preparation of water soluble C-dots was reported by Wu and co-workers [42] by hydrothermal method utilizing the leaves of *Olea europaea* as a carbon source. The as-prepared C-dots with POD-like activity were used for the colorimetric detection of H_2_O_2_ and glucose with detection limits as low as 0.6 μM and 5.2 μM, respectively. The practical utility of this system was also demonstrated for glucose determination in serum samples. This work may pave the way in the exploration of low-cost carbon sources for the synthesis of highly luminescent C-dots using ‘green chemistry’ principles.

In yet another work, high-quality C-dots were prepared from carbonized β-cyclodextrin (β-CD) and investigated as POD mimetics for a sensitive colorimetric assay of H_2_O_2_ with a detection limit of 1 μM. Furthermore, the protocol was successfully extended to develop an excellent analytical platform for oxidative ions, namely, Fe^3+^ and Ag^+^. In particular, the detection limits for Fe^3+^ and Ag^+^ were calculated to be 0.8 and 0.5 μM, respectively [43]. Aside from their frequent use in supramolecular chemistry, this work demonstrate that the special spatial structure of β-CD can also facilitate the formation of high quality C-dots for POD mimic and biosensing.

Very recently, reduced state C-dots have been found to catalyze TMB-H_2_O_2_ system, producing a blue color, a diagnostic for PODs [44]. Based on this finding, a selective colorimetric assay was developed for detection of glucose in human serum samples. Table 1 summarizes the important details of POD-mimicking features of C-dots as discussed above.

## 4. Graphene Quantum Dots (GQDs) as POD Nanozymes

The infinite graphene lattice is a non-luminescent zero-bandgap material with limited practical applications. However, due to their outstanding quantum confinement and edge effect, graphene quantum dots (GQDs) or graphene dots (edge-bound graphene pieces) exhibit bright PL with high photostability and low cytotoxicity, making them an ideal candidate for biochemical sensing and imaging [45].

Li, Yang and co-workers [46] synthesized GQDs by hydrothermal treatment of carbon black (Vulcan XC-72) in the presence of nitric acid. The as-prepared GQDs could effectively catalyze the TMB + H_2_O_2_ system and produced blue coloration in acetate buffer (pH 4.0). By measuring the absorbance changes or monitoring the generation/consumption of H_2_O_2_, the intrinsic POD-like activity of as-prepared GQDs was used in the detection of H_2_O_2_, glucose, and GSH with detection limits of 10 nM, 0.5 μM, and 0.5 μM, respectively. Furthermore, GQDs exhibited high selectivity and was found capable of sensing in complicated biological samples such as cell lysate. The relatively high catalytic activity of GQDs than that of large-sized GO can be attributed to their high diffusion rate and excellent ability to combine with biomolecules.

**Scheme 2:**
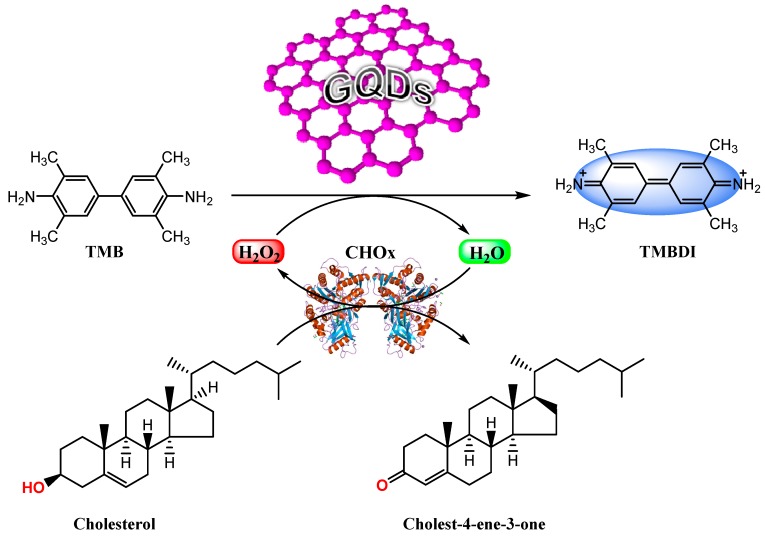
Schematic illustration of GQDs-catalyzed peroxidase mimic and biosensing of cholesterol assisted by cholesterol oxidase (CHOx) and GQDs.

Saxena and co-workers [47] have reported a one-step wet chemical method to generate GQDs directly from graphite. Using TMB as a substrate, the POD-like activity of as-synthesized GQDs was used to detect free cholesterol in human serum with a detection limit of 6 μM (Scheme 2).

In an elegant work, Qu and co-workers [48] have recently provided new insights into the high enzymatic activity of GQDs that may allow one to add critical details to existing catalytic mechanisms with higher confidence. In particular, three GQD derivatives were synthesized through chemical reactions between GQDs and phenyl hydrazine, benzoic anhydride, and 2-bromo-1-phenylethanone as the selective titrants or deactivation agents to respectively react with the ketonic carbonyl, hydroxyl, and carboxylic groups on the GQD’s surface. By measuring the enzyme kinetic parameters of different colloidal GQDs, it was evidenced that the –C=O groups are the main catalytically active sites, whereas the O=C–O– groups serve as the substrate-binding sites. In contrast, the existence of –C–OH group on the surface of GQDs decreases their catalytic activity. The experimental results were further corroborated by theoretical calculations.

## 5. Doped GQDs/C-Dots as POD Nanozymes

Given the remarkable effect of element doping on PL properties of GQDs, Chen and co-workers [49] synthesized nitrogen-doped GQDs (N-GQDs) through strong acid oxidation of three dimensional N-doped graphene aerogel (3D NGA), using dopamine or 3,4-dihydroxyphenethylamine (DA; an important neurotransmitter in mammalian’s brain and body, and a well-known precursor in the synthesis of polydopamine [50]) as a nitrogen source. The as-produced N-GQDs presented intrinsic POD-like activity and could catalyze the TMB + H_2_O_2_ system. The time-dependent absorbance changes at λ 652 nm indicated that with increasing concentration of N-GQDs, the reaction rate and absorbance changes also increase significantly. This may be attributed to the effect of N-doping that can effectively influence the spin density and the charge distribution of carbon atoms, and thus enhances the density of the catalytically active centers on the graphene surface with low stereohindrance for binding redox species in catalytic reactions [51]. The POD-like activity of N-GQDs was also found to be dependent on pH, temperature and H_2_O_2_ concentration, similar to that of HRP. The kinetic studies revealed that the *K*_m_ value of N-GQDs for TMB substrate was 22.9-fold less than that of HRP, indicating that N-GQDs presents a higher affinity to TMB. Using the POD-like catalytic activity of N-GQDs, a selective and quantitative colorimetric assay was developed for detection of H_2_O_2_ and glucose with detection limits of 5.3 μM and 16 μM, respectively. The practical utility of this assay was further demonstrated in glucose detection in diluted serum as well as three commercial fruit juice samples. As a novel natural enzyme mimic, N-GQDs are expected to facilitate the application of the present protocol in medical diagnostics.

Very recently, Wang, Weng and co-workers [52] have synthesized N-doped C-dots (with trace amounts of iron(III)) by hydrothermal treatment of branched polyethylenimine and hemin, an iron-protoporphyrin and natural mimetic enzyme. The POD-like activity of N-doped C-dots has been exploited to develop the both colorimetric and fluorometric assays for DA detection with respective detection limits of 0.4 μM and 20 nM. The assay offers high sensitivity, selectivity and good feasibility in the analysis of DA in human serum even in the presence of multiple interferences except a high concentration of strong reducing agents.

## 6. GQDs/C-Dots Conjugates and/or Nanocomposites as POD Nanozymes

**Figure 5:**
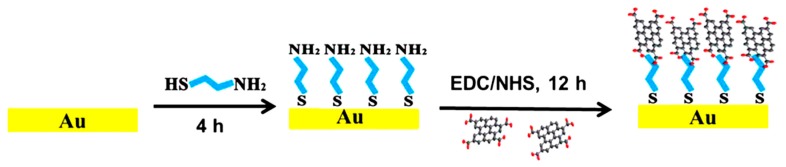
Illustrative fabrication process of the GQDs/Au electrode. Reproduced with permission from [53]. Copyright (2013) Royal Society of Chemistry. Abbreviations: EDC, 1-Ethyl-3-(3-dimethylaminopropyl)carbodiimide; NHS, *N*-hydroxysulfosuccinimide.

In pioneering work, Guo, Zhang and co-workers [53] prepared GQDs by irradiating a mixture of H_2_O_2_ and GO’s suspension using a mercury lamp (365 nm, 1000 W). The as-generated GQDs with an average size of 30 nm produced a blue color of oxidized TMB in the presence of H_2_O_2_. Taking advantage of their structural, electric and excellent POD-like activities (over large-sized GO nanosheets), GQDs were covalently assembled on the surface of gold (Au) to develop a stable enzyme-like electrode. An illustrative fabrication process of the GQDs/Au electrode is shown in Figure 5. The electrochemical measurements revealed that the GQDs/Au electrode can display a fast amperometric response to H_2_O_2_ with a wide linear range (0.002 to 8 mM) and a detection limit down to 0.7 μM. The detection limit was found comparable or even better than many HRP immobilized Au electrodes. Owing to its good electrocatalytic activity, facile preparation, and high stability, GQDs/Au electrode holds great potential as an enzyme-free electrode for sensing assays in pathology and physiology.

**Figure 6:**
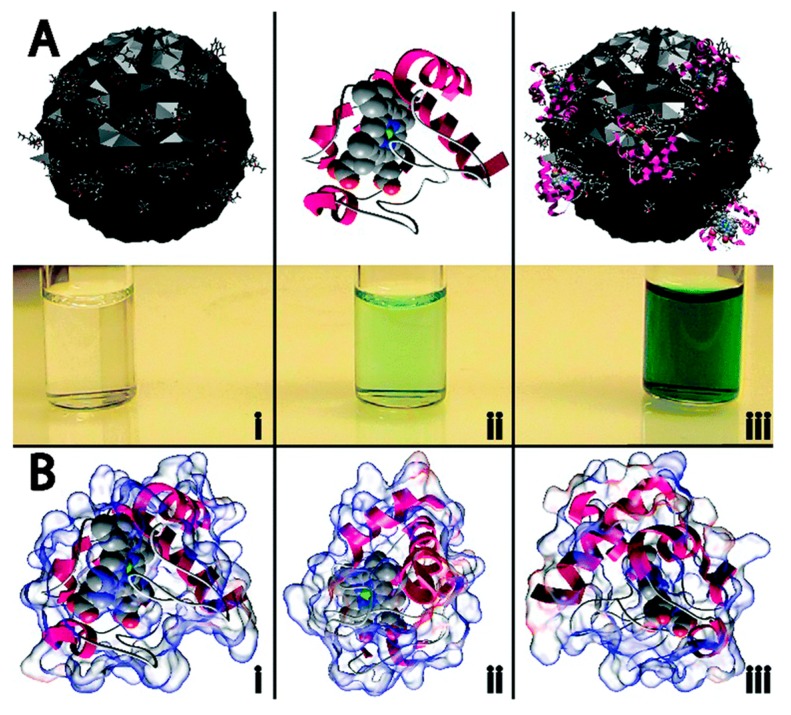
(**A**) Neat carbon nanodots (C-dots) did not display any catalytic activity, as evidenced by the lack of green color in (**Ai**); while native cytochrome *c* (cyt *c*) was found to exhibit weak peroxidase activity (**Aii**); When C-dots were combined with cyt *c*, the peroxidase activity was dramatically enhanced, indicated by the rapid formation of the oxidized form of ABTS (i.e., appearance of intense green color), as shown in (**Aiii**); (**B**) Different orientations of the electrostatic calculations performed on cyt *c* show that a majority of the protein surface is positively charged (blue areas), with the bulk of the positive charge residing in the vicinity of the heme group. Reproduced from [54].

Very recently, Baker and co-workers [54] have reported a straightforward tactic to boost the low POD-like activity of cytochrome *c* (cyt *c*), an electron transfer protein in the respiratory chain, following its electrostatic assembly onto the C-dots surface. Specifically, citric acid (CA) as a carbon source was thermally carbonized alone as well as in the presence of various dopants/passivating agents such as urea (U), thiourea (T) and mercaptosuccinic acid (M), to generate four different CA, CA-U, CA-T, and CA-M-derived C-dots. Figure 6 highlights the schematic postulated interactions between CA-derived C-dots and cyt *c*. Specifically, either only CA-derived C-dots or bare cyt *c* exhibited minimal POD activity as observed by no (Figure 6Ai) or little appearance (Figure 6Aii) of green color in the H_2_O_2_-assisted oxidation of ABTS. However, in the presence of C-dots, the POD activity of cyt *c* enhanced markedly, manifested in a color change from colorless to green (Figure 6Aiii) due to the oxidation of ABTS. The POD activity modulation of cyt *c* (which possesses a largely cationic surface with much of that positive charge residing proximal to the active heme site (Figure 6B)) in the presence of C-dots (having a negatively charged surface due to the presence of a complex cocktail of oxygen-containing functional groups) was attributed to the electrostatic-driven perturbation of heme microenvironment of cyt *c*. The protein conformational changes (i.e., perturbation of secondary structure of cyt *c*) was evidenced using circular dichrosim (CD) measurements, which indicate that an approximate α-helical content of the cyt *c* decreased from an initial value of 24% to just 2.3% upon the addition of CA-derived C-dots. Meanwhile, the random content rose from 34% to 41%. These results indicate that the global protein fold is seriously altered and, in concert with changes local to the heme group, is responsible for the large catalytic enhancement. An exceptional catalytic efficiency (*K*_cat_/*K*_M_) of 8.04 ± 1.74 × 10^7^ M^−1^·s^−1^ was determined for co-assemblies of C-dots/cyt *c*, which were found to be close to the theoretical diffusion-controlled limit. Furthermore, the activity of the C-dots/cyt *c* assembly can be switched off simply by increasing the ionic strength, resulting in dissociation into non-catalytic components. This work suggests the appealing prospects for nanocarbon-based modulation of other potential Fe-centered POD mimics. 

In another work, Qin and co-workers [55] employed positively charged NiAl-layered double hydroxide (NiAl-LDH) nanoplates for electrostatic self-assembly with negatively charged C-dots. Compared to the bare C-dots and NiAl-LDH, self-assembled C-dots/NiAl-LDH exhibited a POD-like activity, producing a color reaction of H_2_O_2_-TMB system. When exposed to different conditions (solvents, temperature), C-dots/NiAl-LDH showed better stability than native HRP and could be used for colorimetric detection of H_2_O_2_ with a detection limit of 0.11 μM. Furthermore, the practical utility of this assay was established for the determination of H_2_O_2_ in milk samples. 

Intrigued by their early work [53], Guo, Zhang and co-workers integrated GQDs with Fe_3_O_4_ NPs via a one-step co-precipitation approach [56]. The as-prepared GQDs-Fe_3_O_4_ composites exhibited excellent POD-like activities than those of individual GQDs, individual Fe_3_O_4_ NPs, and the composites of micrometer-sized GO and Fe_3_O_4_ NPs. The excellent POD-like activities of GQDs-Fe_3_O_4_ composites were attributed to the unique properties of GQDs and the synergistic interactions between the GQDs and Fe_3_O_4_ NPs. Compared to native HRP, the GQDs-Fe_3_O_4_ composite as a catalyst showed better or comparable removal efficiencies for some phenolic compounds from aqueous solutions, with higher stability and reusability. Owing to its superior physical properties in conjunction with catalytic activity, GQDs-Fe_3_O_4_ nanocomposite holds great promise for industrial wastewater treatment.

Yan and co-workers [57] synthesized a ZnFe_2_O_4_-GQDs nanocomposite through a photo-Fenton reaction and demonstrated its POD-like activity in conjunction with HRP, graphene, CNTs, hemin-graphene hybrid, and graphene supported ferric porphyrin. The ZnFe_2_O_4_-GQDs nanocomposite was found to possess higher POD-like activity than each individual, which was attributed to the synergistic effect of the GQDs and ZnFe_2_O_4_. Using the ZnFe_2_O_4_-GQDs as a trace, an electrochemical sensor was fabricated to detect DNA. Under the optimal conditions, the approach provided a wide linear range from 10^−16^ to 5 × 10^−9^ M and low detection limit of 6.2 × 10^−17^ M. As a novel trace label mimic for electrochemical biosensing, ZnFe_2_O_4_-GQDs nanocomposite can be widely used to replace conventional POD-based assays for the identification of other target molecules.

Pt-C-dots nanocomposite constitute the another example of the effective POD mimic based on the synergistic effects between C-dots and Pt NPs [58]. The catalytic efficiency of this composite was found nine and five times higher than those of bare C-dots and Pt NPs, respectively. Mechanistically, the oxidation of TMB was contributed to the production of active oxygen species, in particular, hydroxyl radical by H_2_O_2_ decomposition. Based on the efficient POD-like performance, Pt-C-dots nanocomposite was successfully used for colorimetric detection of H_2_O_2_ and glucose with respective detection limits of 0.8 μM and 1.67 μM. Nevertheless, despite being the robust POD mimic, the use of expensive Pt metal in Pt-C-dots nanocomposite somewhat limits the practical utility of this protocol.

**Figure 7:**
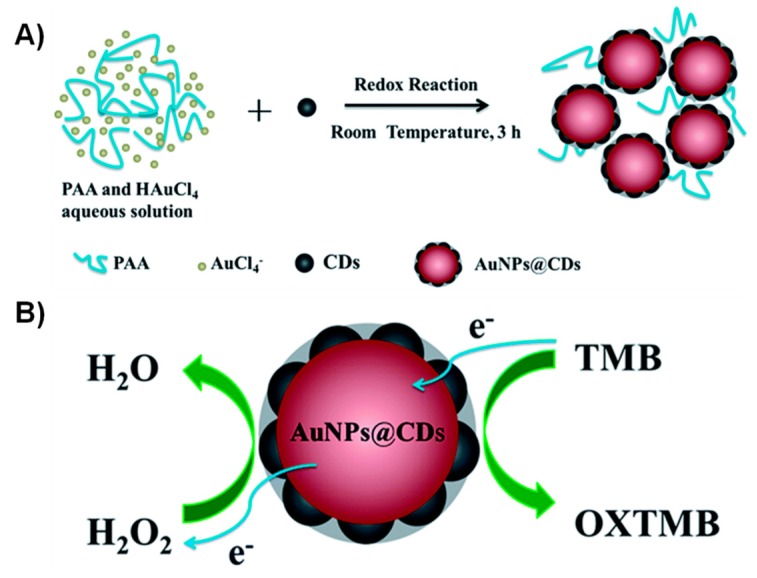
(**A**) The Schematic illustration of the reaction process; (**B**) Proposed mechanism over Au NPs@C-dots nanocomposite as POD mimic. Reprinted with permission from ref. [59]. Copyright (2016) Royal Society of Chemistry.

Very recently, An and co-workers [59] have synthesized well-dispersed Au NPs@C-dots nanocomposites (with core-shell nanostructure and the ultrathin carbon layer of ca. 1-2 nm) by C-dots-induced reduction of chloroauric acid at room temperature (Figure 7A). The Au NPs@C-dots composites are found to possess intrinsic POD-like activity, and the catalytic activity is higher than those of HRP and bare Au NPs. The mechanism of POD-like activity of Au NPs@C-dots was extensively investigated by a combination of fluorescence spectroscopy, electron spin resonance (ESR), and cyclic voltammetry (CV) and could be ascribed to the facile electron transfer between TMB and H_2_O_2_ (Figure 7B). Due to their good catalytic activity and biocompatibility, Au NPs@C-dots may pave their way in the fields of biotechnology and clinical diagnosis.

## 7. Carbon Nitride Dots (CN-Dots) as POD Nanozymes

In an elegant work, Sun and co-workers [60] reported a general strategy for the production of photoluminescent carbon nitride dots (CN-dots) based on the microwave heating of a variety of organic amines such as dimethylamine, ethylamine or tripropylamine, in the presence of different mineral acids including chlorosulfonic acid. The resultant CN-dots were found to possess POD-like activity as inferred by the blue color reaction of TMB-H_2_O_2_ system. The practical application of CN-dots as POD mimic was demonstrated in the colorimetric detection of H_2_O_2_ and glucose with detection limits of 0.4 μM and 0.5 μM, respectively. Furthermore, the CN-dots-based glucose biosensing system exhibited excellent selectivity over fructose, lactose, and maltose. Table 2 summarizes the important details of POD-mimicking features of GQDs/C-dots conjugates or nanocomposites and CN-dots as described below.

## 8. Advantages, Challenges and Future Perspectives

In order to fully realize the potential of C-dots domain as POD nanozymes, it would be worthwhile to pinpoint their key advantages or favorable features over noble metal/metal oxide nanomaterials. Specifically, diversified synthesis, cost-effectiveness, excellent water-dispersibility, (photo)chemical stability, photoluminescent properties, low toxicity/biocompatibility, and most importantly, on-demand surface engineering are the remarkable advantages of C-dots, formulating them as the materials-of-choice for POD mimetics and biosensing applications. Despite this, in contrast to metal/metal oxide NPs or materials, C-dots suffer with certain disadvantages or challenges such as low product yield, difficulties associated with their purifications, which, in turn, do not allow their large-scale production, and the deficiencies in precisely controlling the uniform size of C-dots, a factor that may significantly influence the POD-like activity of C-dots. Not surprisingly, therefore, despite the overwhelming collection of reports on carbonaceous nanomaterials, in particular, 2D graphene with POD-like activities, the research progress on C-dots as POD nanozymes is still in its infancy, leaving a wide open space for future developments. Taking into account the recent advancements in the field as well as dictated by future applications, we speculate that the significant trends in C-dots-based POD mimic should follow the development of high performance synergistic nanocomposites, functional assemblies for cascade reactions, and a variety of doped C-dots. Manifestation of such improvements will possibly lead to the development of inexpensive, portable, and conductive to commercialization C-dots-based test strips as practical kits for sensing purposes as well as may significantly influence the current biotechnology and bioanalytical chemistry in the context of point-of-care detection.

## 9. Conclusions

The aim of this mini review has been to illustrate the many carbon-based nanodots and composites or systems that have been utilized as effective POD nanozymes for biosensing applications. As shown here, considerable attention has been paid on the development of sensitive and selective colorimetric assay to detect glucose in biological as well as real samples. The other indirect approaches, employing carbon-based nanodots as a ‘key’ in either synthesis [61] or to detect toxic and environmentally questionable analytes such as dihydroxybenzene [62] and bisphenol A [63], whilst beyond the scope of this review, certainly, dictate the efficacy of nanodots in the context of POD mimics. Given the fast pace of this area along with the exceptional possibility for on-demand surface engineering of carbon-based nanodots, one thing for sure that carbon-based nanodots as POD nanozymes have tremendous potential and will continue to thrive in near future for biosensing, biotechnology, clinical diagnostics, and other industrial applications.

## Figures and Tables

**Table 1:** POD-like activity of carbon nanodots. ^a^

Nanomaterial(s)	Synthesis Method	Substrate(s)	Application(s)	LOD	Reference
C-dots ^b^	Oxidation with HNO_3_	TMB, OPD, and THB	H_2_O_2_ detection	0.2 μM	[36]
			Glucose detection	0.4 μM	
CNPs ^c^	Oxidation with HNO_3_	TMB and DAB	H_2_O_2_ detection	1.0 μM	[37]
			Glucose detection	20.0 μM	
C-dots	Electrochemical	−	Degradation of MO azo dye	−	[38]
CDs ^d^	MW-IL method	−	Degradation of MO and MR azo dyes	−	[39]
CDs ^d^	Hydrothermal	TMB	GSH detection	0.3 μM	[40]
CDs ^d^	Hydrothermal	TMB	Hg^2+^ detection	23 nM	[41]
CDs ^d^	Hydrothermal	TMB	H_2_O_2_ detection	0.6 μM	[42]
			Glucose detection	5.2 μM	
CDs ^d^	Calcination followed by oxidation with HNO_3_	TMB	H_2_O_2_ detection	1.0 μM	[43]
			Fe^3+^ detection	0.8 μM	
			Ag^+^ detection	0.5 μM	
r-CDs ^d^	Oxidation with HNO_3_	TMB	Glucose detection	2.0 μM	[44]

^a^ C-dots, Carbon nanodots; LOD, Limit of detection; TMB, 3,3’,5,5’-tetramethylbenzidine; OPD, *o*-phenylenediamine; THB, 1,2,3-trihydroxybenzene; CNPs, Carbon nanoparticles; DAB, Diazoaminobenzene; MW-IL, Microwave-assisted ionic liquid; GSH, Glutathione; ^b^ Also exhibit catalase-mimicking activity; ^c^ CNPs represent C-dots as per ref. [37]; ^d^ CDs represent C-dots as per refs. [39,40,41,42,43,44].

**Table 2:** POD-like activity of GQDs/C-dots conjugates or nanocomposites and carbon nitride (CN) dots ^a^.

Nanoconjugate(s)/nanocomposite(s)	Synthesis Method	Substrate(s)	Application(s)	LOD	Reference
GQDs/Au	Photo-Fenton reaction and covalent assembly	TMB	H_2_O_2_ detection	0.7 μM	[53]
C-dots/cyt *c*	Thermal pyrolysis and electrostatic assembly	ABTS	Enzyme modulation	−	[54]
C-dots/NiAl-LDH	Electrostatic assembly	TMB	H_2_O_2_ detection	0.11 μM	[55]
GQDs-Fe_3_O_4_	Co-precipitation	TMB	Removal of phenolics	−	[56]
ZnFe_2_O_4_-GQDs	Hydrothermal + photo-Fenton reaction	TMB	DNA detection	62 *a*M	[57]
CDs-Pt ^b^	Hydrothermal + mechanical stirring	TMB	H_2_O_2_ detection	0.8 μM	[58]
			Glucose detection	1.67 μM	
Au NPs@CDs ^b^	MW-assisted synthesis + chemical reaction	TMB	−	−	[59]
CNDs ^c^	MW-assisted synthesis	TMB	H_2_O_2_ detection	0.4 μM	[60]
			Glucose detection	0.5 μM	

^a^ GQDs, Graphene quantum dots; C-dots, Carbon nanodots; LOD, Limit of detection; GQDs/Au, Covalently assembled GQDs on the Au electrode surface; TMB, 3,3’,5,5’-tetramethylbenzidine; cyt *c*, cytochrome *c*; ABTS, 2,2′-azino-bis-(3-ethylbenzothiozoline-6-sulfonic acid; C-dots/NiAl-LDH, Non-covalently assembled C-dots with positively charged NiAl-layered double hydroxide nanoplates; NPs, Nanoparticles; GQDs-Fe_3_O_4_, GQDs integrated with iron oxide NPs; ZnFe_2_O_4_-GQDs, GQDs assembled on the magnetic ZnFe_2_O_4_; *a*M, Attomolar (10^−18^ M); CDs-Pt, C-dots-Pt NP composites; Au NPs@CDs, Core-shelled Au NPs@C-dots nanocomposites; MW, Microwave; CNDs, Carbon nitride dots; ^b^ CDs represent C-dots as per refs. [58,59]; ^c^ CNDs represent CN-dots as per ref. [60].
